# Total Score of Athleticism: Profiling Strength and Power Characteristics in Professional Soccer Players After Anterior Cruciate Ligament Reconstruction to Assess Readiness to Return to Sport

**DOI:** 10.1177/03635465231194778

**Published:** 2023-09-08

**Authors:** Luca Maestroni, Anthony Turner, Konstantinos Papadopoulos, Vasileios Sideris, Paul Read

**Affiliations:** † ReAct, Bergamo, Italy; ‡ London Sport Institute, Faculty of Science and Technology, Middlesex University, London, UK; § School of Allied Health and Community, University of Worcester, Worcester, UK; ‖ Rehabilitation Department, Aspetar Orthopaedic and Sports Medicine Hospital, Doha, Qatar; ¶ Institute of Sport, Exercise and Health, London, UK; # School of Sport and Exercise, University of Gloucestershire, Gloucester, UK; ** Division of Surgery and Interventional Science, University College London, London, UK; †† Faculty of Sport, Allied Health and Performance Sciences, St Marys University, Twickenham, UK; Investigation performed at Aspetar Orthopaedic and Sports Medicine Hospital, Doha, Qatar

**Keywords:** ACL reconstruction, athleticism, soccer, return to play

## Abstract

**Background:**

There is no consensus on the optimal testing procedure to determine return-to-sport (RTS) readiness after anterior cruciate ligament (ACL) reconstruction. Current approaches use limb symmetry across a range of tests, but this does not consider a patient’s level of athleticism or benchmarks relative to his or her noninjured counterparts.

**Purpose:**

To examine the utility of the Total Score of Athleticism (TSA), a composite scale including strength, power, and reactive strength assessments, to aid RTS decision-making.

**Study Design:**

Cross-sectional study; Level of evidence, 3.

**Methods:**

A total of 95 professional soccer players (60 who underwent ACL reconstruction [mean age, 25.1 ± 12.6 years] and 35 who were uninjured [mean age, 23.8 ± 2.8 years]) completed a battery of tests including isokinetic knee extension and flexion torque, bilateral and unilateral countermovement jump height, relative peak power, and reactive strength index–modified. The TSA score (derived from *Z* scores) was calculated, and we (1) examined differences between the ACL-reconstructed and uninjured groups at the time of RTS, (2) assessed the predictive ability of the TSA to identify the player’s status (ACL reconstruction vs uninjured control), and (3) included a case series to discuss the characteristics of players who sustained a subsequent injury within 4 months after RTS.

**Results:**

A large difference between the ACL-reconstructed and uninjured groups in the TSA score (*d* = 0.84; *P* < .0001) was evident. For every additional increase of 1 unit in the TSA score, the odds of belonging to the ACL-reconstructed group decreased by 74% (95% CI, 0.19-0.56). By visual inspection, the frequency of reinjured players was higher in the low (4/7) TSA tertile compared with the medium (2/7) and high (1/7) TSA tertiles.

**Conclusion:**

Preliminary evidence indicates that the TSA may be a useful RTS readiness tool, as the composite score derived from strength and power measures was different in soccer players at the time of RTS after ACL reconstruction compared with healthy matched controls. There was also a higher frequency of low TSA scores in players who sustained a second injury after RTS. Therefore, it is recommended to routinely administer RTS tests encompassing strength, power, and reactive strength qualities each season across the largest possible number of players (ideally teammates).

At 1 year after anterior cruciate ligament (ACL) reconstruction, most elite soccer players return to play (>90%)^8,49^; however, only two-thirds compete at the same preinjury level 3 years later.^30,49,55^ An ACL injury has been associated with cartilage compositional changes and early joint degeneration in young patients,^20,21,43^ and having undergone ACL reconstruction is a risk factor for future injuries in athletes participating in multidirectional field sports (odds ratio, 2.2 [95% CI, 1.1-4.4]; *P* = .029).^28^ Elite soccer players who have undergone ACL reconstruction also display a nearly 20-fold increased risk of sustaining a subsequent ipsilateral or contralateral rupture in comparison to matched healthy players.^30^ Reduced reactive strength^15^ and knee extension strength^10^ are modifiable risk factors associated with a secondary injury.

Male soccer players who have undergone ACL reconstruction display reduced strength, power, and reactive strength absolute values in the operated limb in comparison to healthy controls.^24,31,38,39^ Assessments of these fundamental physical characteristics can help practitioners to quantify neuromuscular qualities that underpin movements inherent to soccer such as sprinting, jumping, and change of direction.^12,14^ Owing to different multidimensional aspects involved with return to sport (RTS),^7^ there is no consensus on when an athlete is ready to RTS or the optimal testing procedure to determine sport readiness.^3^ Current practice^48^ involves a battery of strength and hop tests, with a limb symmetry index of ≥90% recommended as the cut-off point to determine “pass” or “fail.”^4,19^ However, this does not consider potential performance decrements in the uninvolved limb after an injury and surgery, thus limiting the utility of this approach.^4,19^ Research has shown that a low proportion of patients (23%) pass these RTS criteria (ie, based on symmetry scores ≥90%) but do still return to play.^50^ Furthermore, only a minority of noninjured athletes pass these tests, meeting the ≥90% symmetry criteria (~24%).^26^ This may be because of the reduction in the probability of passing when multiple tests across a number of domains are added to a battery, limiting the utility of this approach for the purpose of augmenting RTS decision-making.^50^

Rather than separately analyzing each individual test result using predetermined symmetry thresholds, a composite score encompassing different performance characteristics can be calculated for each player. This approach has already been adopted in fitness testing, using standardized scores from a series of tests to create a single Total Score of Athleticism (TSA) value for each individual player.^45^ By averaging standardized scores (ie, *Z* scores) and applying the TSA instead of only interlimb symmetry in different tests, this allows clinicians and coaches to examine contextualized data of individual athletes relative to their teammates and thus set benchmarks for RTS readiness that are realistic to the demands that athletes will be exposed to. Oleksy et al^32^ showed reduced composite scores (using Functional Movement Screen, Y-Balance Test, and tuck jump assessment) in Polish players who underwent ACL reconstruction in comparison to healthy controls. However, these instruments do not primarily examine the physical characteristics underpinning athletic movements related to the injury risk. The utility of this novel approach using absolute strength and power qualities has yet to be examined in athletic populations aiming to RTS after ACL reconstruction and the completion of rehabilitation.

This study aimed to (1) investigate if there are differences in the TSA score between the ACL-reconstructed and uninjured groups; (2) examine the predictive ability of the TSA to identify group membership (ACL reconstruction vs healthy control); and (3) include a case series to discuss the characteristics of players who, having undergone ACL reconstruction, sustained a subsequent injury within 4 months after RTS.

## Methods

### Participants

A total of 60 male soccer players participating in the Qatar Stars and Qatargas Leagues (mean age, 25.1 ± 12.6 years; mean height, 175.8 ± 9.2 cm; mean weight, 74.3 ± 14.0 kg), with a mean of 9.2 ± 3.0 months after ACL reconstruction, volunteered to take part in this study. The majority of ACL grafts were bone–patellar tendon–bone grafts (80%), with the remaining players (20%) receiving hamstring tendon (semitendinosus and gracilis) grafts. Inclusion criteria required players to have no previous ACL injuries/surgery or other knee ligament or cartilage injuries/surgery in either the operated or nonoperated leg. All participants were involved in an intensive supervised rehabilitation program (5 d/wk) at the same sports medicine hospital,^19^ which commenced immediately after surgery; they were required to have completed the early, intermediate, and advanced phases of rehabilitation and be active in on-field, sport-specific rehabilitation exercises. The focus of the early phase was controlling swelling, restoring range of motion, and activation of the knee extensor and flexor muscles. The goal of the intermediate and advanced phases was to optimize muscle strength, proprioception, and neuromuscular control and complete a phased-progression running program. After the completion of these phases, players took part in an on-field, sport-specific training and conditioning block. Informed written consent was obtained before participation.

We also recruited 35 (uninjured) matched controls (mean age, 23.8 ± 2.8 years; mean height, 173.8 ± 5.4 cm; mean weight, 71.6 ± 6.3 kg) from the same leagues, who underwent preseason screening at a national sports medicine institution and were randomly selected from a pool of 300 athletes. Inclusion was based on having no history of ACL injuries and being free from any severe injury (defined as time loss >28 days) in the previous 12 months, verified via a national injury audit. Clubs competing in the stated leagues within Qatar regularly undergo formalized strength and conditioning training including resistance, speed, agility, and plyometric exercises. Data for the players who underwent ACL reconstruction were collected from 2017 to 2020, and data for the healthy controls were collected in 2017 at the onset of the study. This study was approved by an institutional review board (No. F2017000227) and research ethics committee (No. 14326).

### Experimental Design

To address our stated aims, we (1) calculated the TSA score using standardized scores of performance variables obtained from isokinetic strength assessments (ie, knee extension and flexion relative peak torque of both limbs) and from double- and single-leg countermovement jump (CMJ) tests (ie, jump height, relative peak power, and reactive strength index–modified [RSImod]) and then compared the TSA score between the ACL-reconstructed and uninjured groups; (2) examined the ability of the TSA to identify group membership (ACL-reconstructed or uninjured group); and (3) completed a case series of players who had further injuries in the first 4 months after rehabilitation and RTS. This time period was chosen to avoid the confounding effects of regular soccer training and seasonal variations on strength and power characteristics.^1^

All participants were familiar with the testing procedures and completed a standardized warm-up consisting of 5 minutes of pulse-raising activity (stationary cycling performed at 60% of maximum perceived effort), followed by 10 body-weight squats (bilateral and unilateral), lunges, and step-ups. CMJs were then completed at 50%, 75%, and 90% of perceived maximum.^37^ Isokinetic assessments were undertaken after the jump tests. Assessments were conducted under the supervision of an experienced investigator (>5 years using the stated test methodology).

### Injury Reporting

The orthopaedic and sports medicine hospital involved in this study provides medical and sports science services to all sports clubs in the country. As part of this program, it is mandatory to report any injuries that occur in players on their hospital medical record. Furthermore, a national injury audit is performed annually and coordinated by the hospital’s research department. The research department also employs a research assistant to work as part of the ACL assessment pathway, who contacts all players for routine follow-up every 3 months after RTS. Injuries were recorded if they resulted in time loss from their sport, and all were confirmed via magnetic resonance imaging at the same orthopaedic and sports medicine hospital. A time-loss injury was classified as an occurrence resulting in days lost from training sessions and matches.

### Testing Procedures

#### Isokinetic Knee Extension and Flexion Strength

Maximum quadriceps extension peak torque and hamstring flexion peak torque relative to body weight (N·m/kg) were measured using an isokinetic dynamometer (Biodex Medical Systems). Players were in a seated position with the hip flexed to 90°. Then, 5 repetitions of concentric knee extension and flexion were performed at 60 deg/s with the highest peak torque value recorded.^47^ Peak torque values were reported as a percentage of the player’s body weight. Procedures were explained to participants, after which they completed 3 practice repetitions; testing then commenced after 60 seconds. Limb order was randomized. Standardized, vigorous verbal encouragement was provided throughout. No formal familiarization session was provided, but all participants had previous experience in isokinetic testing with regular monitoring throughout their rehabilitation process.

#### Countermovement Jump (Bilateral/Unilateral)

Participants were instructed to stand fully upright, with hands on hips, and align their feet on a synchronized, uniaxial dual force plate system (ForceDecks Version 1.2.6109; VALD Performance). Before the initiation of the test, each player was instructed to remain motionless for a minimum of 3 seconds to ensure that a stable baseline of force at body weight was obtained. Players then performed a downward motion (descent phase) until they reached their preferred self-selected depth^34^ before rapidly reversing the motion by triple extending at the hip, knee, and ankle. The aim of the task was to achieve their maximum vertical displacement of the center of mass. Hands remained on the hips throughout, and no bending of the knees was permitted while airborne. The procedures were replicated for the single-leg CMJ, except that the nontested leg was positioned with the hip and knee at 90° and no obvious swinging was allowed to minimize contralateral propulsion. Limb order was randomized. There were 2 trials performed with a 30-second rest period between each jump, with the best trial recorded for statistical analysis.

Vertical ground-reaction force data were recorded at a sampling rate of 1000 Hz. The initiation of the jump was defined by a 20-N change in body weight calculated between the quiet standing period and the instant of takeoff, when the total vertical force dropped below 20 N. Jump height was calculated from the impulse-momentum relationship–derived take-off velocity and the equation for constant acceleration (velocity at takeoff squared divided by 2 * 9.81 and [v^2^/2g]). Peak power was measured and normalized to body weight (W/kg; relative peak power) during the propulsion phase. The RSImod value was calculated by dividing jump height by contraction time (determined from movement onset to time to takeoff).^44^ This variable was used to determine the ability to store and reutilize elastic energy during stretch-shortening cycle activities.^9^

#### Total Score of Athleticism

A composite score of physical capacity was derived for each player by averaging standardized scores from knee extension and flexion relative peak torque of both limbs, double- and single-leg CMJ height, relative peak power, and RSImod. To calculate the *Z* score of each test, the following formula was used: *Z* score = (player score – cohort mean) / cohort standard deviation. Finally, the TSA score was calculated by averaging all *Z* scores.^45^

The TSA is a measure used across sports and performance settings,^36,54^ including athletes returning after ACL reconstruction.^32^ The use of *Z* scores allows clinicians to compare data across similar athletes, who share the same training approach, demands, and constraints. Therefore, test scores are assumed achievable by all athletes and thus represent realistic targets and thresholds that can be worked toward. Therefore, to define these benchmarks, injured athletes must be measured alongside their healthy teammates (matched controls). Furthermore, it should be noted that the TSA score (and all individual *Z* scores) is a relative score that cannot be applied to a different group and thus compared across sports and previously published normative tables. Instead, the TSA defines how an athlete ranks among his teammates, who are similarly affected by a club’s training philosophy and resources, and may highlight injured athletes who still display performance decrements and are thus not ready to RTS. Finally, the TSA is a composite scale of the chosen tests, which is further influenced by the weighting of those tests. For example, more tests may be included that measure strength than endurance, and thus, the TSA score will have a bias toward strength. The tests must therefore be chosen appropriately and are likely based on the experience of the clinicians and the type of injury. In summary, the TSA is specific to the tests chosen as well as the group tested, whereby a deviation from the mean (represented by 0), which is expressed as the standard deviation, is likely to be the only transferable value that may be applied to other clinical practices.

### Statistical Analysis

The distribution of the data was assessed using the Kolmogorov-Smirnov normality test. Descriptive statistics (mean ± standard deviation) for all variables were calculated.

An independent-samples *t* test was used to examine differences in demographic characteristics and TSA scores between the ACL-reconstructed and uninjured groups. The Cohen *d* (effect size) with the 95% confidence interval was calculated to interpret the magnitude of these differences: standardized mean differences of 0.2, 0.5, and 0.8 indicated small, moderate, and large effect sizes, respectively.

Binary logistic regression was used to examine the predictive ability of the TSA in identifying group membership (ACL-reconstructed or uninjured group). Unstandardized coefficients (β) and adjusted *R*^2^ values were reported. Odds ratios were calculated via logistic regression with 95% confidence intervals. Statistical significance was set at *P* < .05. All data were computed through Excel (Version 2010; Microsoft). Data processing was performed, and descriptive statistics were determined, using SPSS (Version 25; IBM). Given the sampling procedure for such research, post hoc power analysis using G*Power (Version 3.1.9.7) revealed that the statistical power for within-group comparisons was 96% for detecting a large effect size, 64% for a moderate effect size, and 15% for a small effect size.

TSA results were divided into tertiles (tertile 1 = low; tertile 2 = medium; and tertile 3 = high) using visual inspection of the distribution, performance characteristics, and clinical history of ACL-reconstructed players who had further injuries within 4 months after RTS.

## Results

The TSA score was significantly lower in the ACL-reconstructed group compared with the uninjured group (*d* = 0.84 [95% CI, 0.40-1.27]; *P* < .0001) (Table 1). Logistic regression analysis showed that the TSA score accounted for 20% of the variability observed in group membership (*R*^2^ = 0.200). For every additional increase of 1 unit in the TSA score (β = −1.357), the odds of belonging to the ACL-reconstructed group decreased by 74% (95% CI, 0.19-0.56).

**Table 1:** Differences in Characteristics Between ACL-Reconstructed and Uninjured Groups^*a*^

	ACL-Reconstructed Group (n = 60)	Uninjured Group (n = 35)	Cohen *d* (95% CI)	*P* Value
Age, y	25.1 ± 12.6	23.8 ± 2.8	0.12 (–0.30 to 0.53)	.578
Height, cm	175.8 ± 9.2	173.8 ± 5.4	0.25 (–0.17 to 0.67)	.180
Weight, kg	74.3 ± 14.0	71.6 ± 6.3	0.23 (0.19 to 0.65)	.199
TSA score	−0.20 ± 0.76	0.35 ± 0.43	0.84 (0.40 to 1.27)	<.0001
Time from surgery, mo	9.2 ± 3			
Reinjury within 4 mo, n	7			
Subsequent injuries	Grade 2 medial femoral condyle cartilage injury, grade 2 rectus femoris injury (n = 2), grade 1 rectus femoris injury, grade 1 biceps femoris injury, bucket-handle medial meniscal tear, deep chondral fissure in knee, grade 2 distal myotendinous junction biceps femoris injury (n = 2), bone edema in knee, lateral meniscal tear, medial meniscal tear, parameniscal cyst			

a Data are shown as mean ± SD unless otherwise indicated. Significant difference between groups: *P* < .05. ACL, anterior cruciate ligament; TSA, Total Score of Athleticism.

**Figure 1.**
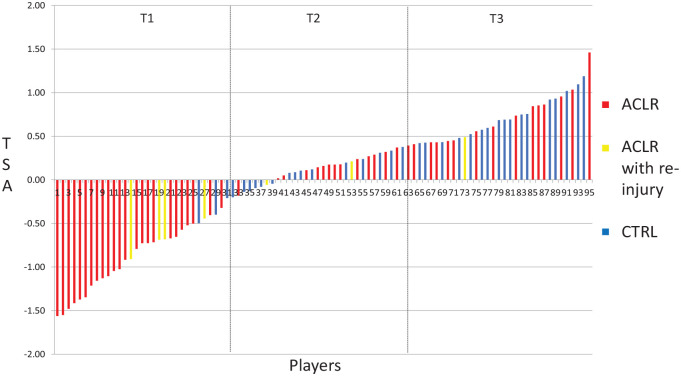
Total Score of Athleticism (TSA) values of anterior cruciate ligament (ACL)–reconstructed players who did not suffer from reinjuries (ACLR), ACL-reconstructed players who suffered from reinjuries (ACLR with reinjury), and healthy controls (CTRL). T, tertile.

Of the 60 included players who had undergone ACL reconstruction, 7 suffered a further injury within 4 months after RTS. The distribution of reinjured players is graphically represented in Figure 1. The frequency of players who sustained a reinjury was higher in the low (4/7) TSA tertile compared with the medium (2/7) and high (1/7) TSA tertiles (observed using visual inspection). From the 7 players identified (mean RTS, 8.8 ± 1.7 months), a total of 13 subsequent injuries were documented. Among these, 5 included articular cartilage and meniscal injuries, whereas the remaining 8 were classified as soft tissue injuries. No ipsilateral or contralateral ACL injuries were documented during our selected time period.

## Discussion

The aims of this study were to (1) investigate differences in the TSA score (derived from strength and power measures) between players who had undergone ACL reconstruction and those competing at the same level of play who were uninjured; (2) examine the association between TSA scores and group membership (ACL reconstruction vs uninjured control); and (3) complete a case series using the TSA among players with ACL reconstruction who had further injuries within 4 months after RTS.

The results showed that the TSA score was substantially lower in players who had undergone ACL reconstruction than healthy controls at the time of RTS (*d* = 0.84 [95% CI, 0.40-1.27]). Lower scores for the examined physical qualities (ie, strength, power, and reactive strength) have been associated with reduced performance in more complex athletic skills, such as pivoting, cutting, landing, and jumping, which are critical to soccer athleticism and RTS.^5,6^ Using the TSA to determine physical readiness may overcome some of the limitations associated with RTS testing. First, the TSA avoids the need for passing tests using symmetry alone, reducing the overestimation of recovery rates (using the potentially deteriorated contralateral limb),^29,53^ and includes comparative data from matched healthy controls. Furthermore, this approach avoids the normal reduction in the probability of passing when there is a requirement to obtain a specific score across multiple tests. Importantly, the TSA allows the determination of single test scores within a general measure of performance level instead of binary “pass” or “fail” criteria. This permits the contextualization of a single player’s data in relation to his teammates and can be used to set benchmarks and rehabilitation goals that are realistic during rehabilitation for the restoration of physical performance to a level no less than that of uninjured players and are reflective of RTS demands.^45^

Regression analysis showed that the TSA score accounted for 20% of the variability observed in the identification of a player’s status. Although the optimal testing procedure to determine sport readiness is currently unclear,^3^ our results confirm the utility of an overall measure of contextualized physical preparedness before RTS to differentiate between injured and uninjured players. Indeed, the odds of shifting toward a healthy player’s profile increase with improvements in the TSA score. For every 0.5 increase in the TSA score, the odds of belonging to the ACL-reconstructed group decreased by 49%, and an increase of 1 unit decreased the odds of being in the ACL-reconstructed group by 74%. To understand which specific component of the total score needs specific attention, each physical characteristic can be broken down and further analyzed by using *Z* scores and respective threshold values (Table 2).

**Table 2:** Physical Characteristics Threshold for our Cohort in each Tertile^*a*^

Tertile	CMJ Jump Height (cm)	CMJ Rel Peak Power (W/kg)	CMJ RSImod (m/s)	SLCMJ Height UNINV (cm)	SLCMJ Rel Peak Power UNINV (W/kg)	SLCMJ RSImod UNINV (m/s)	SLCMJ Height INV (cm)	SLCMJ Rel Peak Power INV (W/kg)	SLCMJ RSImod INV (m/s)	Rel Knee Extension Strength UNINV (Nm/kg)	Rel Knee Extension Strength INV (Nm/kg)	Rel Knee Flexion Strength UNINV (Nm/kg)	Rel Knee Flexion Strength INV (Nm/kg)	TSA
First	<33.5	<47.4	<0.39	<16.1	<29.3	<0.18	<14.0	<27.5	<0.16	<3.0	<2.8	<1.6	<1.6	<–0.20
Second	33.5 to 36.3	47.4 to 52.6	0.39 to 0.47	16.1 to 19.2	29.3 to 33.0	0.18 to 0.24	14.0 to 17.5	27.5 to 30.6	0.16 to 0.21	3.0 to 3.4	2.8 to 3.1	1.6 to 1.9	1.6 to 1.9	−0.20 to 0.39
Third	>36.3	>52.6	>0.47	>19.2	>33.0	>0.24	>17.5	>30.6	>0.21	>3.4	>3.1	>1.9	>1.9	>0.39

a CMJ, countermovement jump; RSImod, reactive strength index–modified; TSA, Total Score of Athleticism.

**Figure 2.**
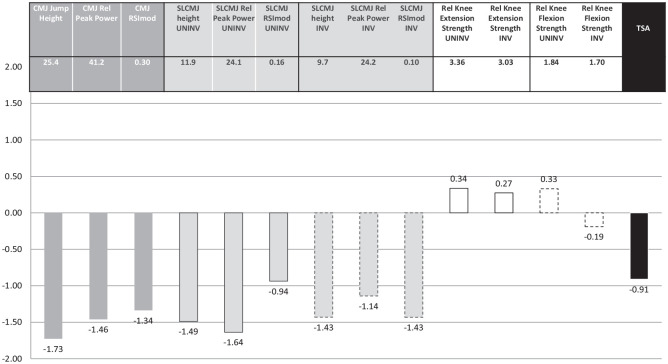
Player 14’s strength, power, and reactive strength values and standardized scores.

**Figure 3. fig3-03635465231194778:**
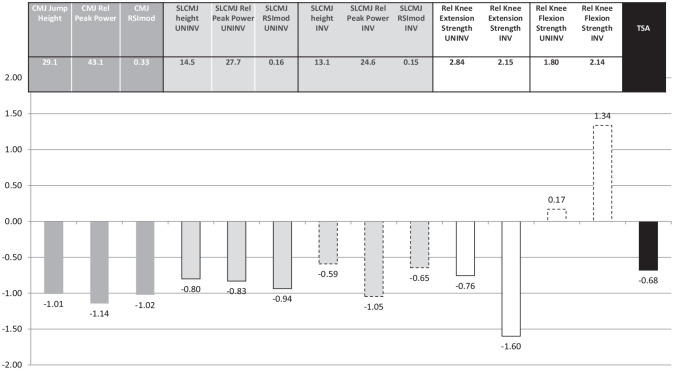
Player 20’s strength, power, and reactive strength values and standardized scores.

Data visualization using a simple schematic (Figures 2 [Fig fig3-03635465231194778]-4) can be a logical and simple way to understand the weaknesses and strengths of each individual player (ie, scores <0 or >0 indicating an athlete being worse or better than average) and can be used to identify one or multiple components to be targeted to collectively increase the TSA score during specific rehabilitation and training cycles.^45^ Pragmatically, bars below zero represent opportunities for improvement that should be targeted during rehabilitation before RTS to achieve important, safe, and specific physical quality thresholds and to increase the TSA score overall.

**Figure 4.**
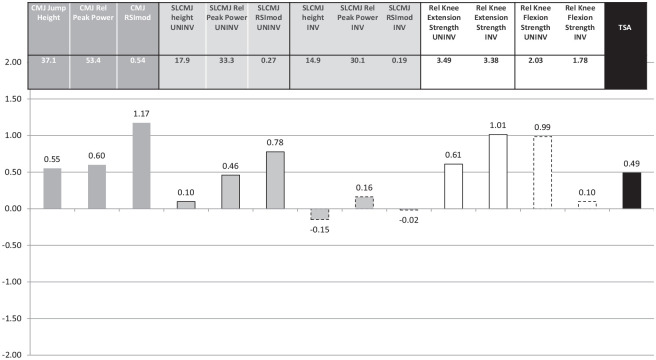
Player 73’s strength, power, and reactive strength values and standardized scores.

### Case Series Analysis

ACL injuries can have a detrimental effect on individual athletic performance, and this may increase the risk of subsequent injuries.^28,30^ Current evidence indicates equivocal findings that passing current RTS criteria is associated with a reduction in the risk of subsequent ipsilateral or contralateral ACL ruptures.^2,19,22,50,52^ The preliminary findings of our case series showed that among the 7 players who sustained a further injury within 4 months after RTS, only 1 player was in the high TSA tertile. The other players were either in the low (4/7) or medium (2/7) TSA tertile, suggesting that higher composite scores encompassing strength and power qualities may be protective toward further articular cartilage, meniscal, and soft tissue injuries at the time of RTS. We elected to conduct a case series, as our sample size was not large enough to examine these associations using regression analysis.

To demonstrate the practical utility of the TSA, we compared 3 players and also determined how a targeted test-training integration process can be used to optimize readiness to RTS. Player 14 (18th percentile; TSA score = −0.91; 30 years old; 166 cm; 58 kg; hamstring tendon graft; 9.5 months after surgery) (Figures 1 and 2) displayed lower power (double-leg CMJ: relative peak power = 41.2 W/kg, jump height = 25.4 cm) and reactive strength (double-leg CMJ: RSImod = 0.30) characteristics within our cohort. Also, jump height and relative peak power did not meet currently available reference values (ie, jump height = 34.5 ± 4.0 cm, relative power = 50.4 ± 4.9 W/kg).^39^ In addition, he showed lower relative peak knee flexion strength (1.70 Nm/kg) of the ACL-reconstructed limb compared with the rest of the group. At about 2 months after RTS, he was diagnosed with a deep chondral fissure and distal biceps femoris myotendinous junction strain injury in the involved knee. Maladaptive functioning of the dampening mechanisms has been demonstrated after ACL reconstruction.^40^ This can impair force attenuation during fast sporting actions such as jumping, landing, and change of direction, exposing athletes to large impact forces, which have been associated with more deleterious compositional changes in articular cartilage of the tibiofemoral compartment.^35^ Similarly, athletes with a history of ACL reconstruction and lower knee flexor strength have a higher probability of future hamstring strain injuries than stronger athletes,^28^ and knee flexor strength deficits are more pronounced in those who receive a hamstring tendon graft.^24^ For this player, it seems reasonable to suggest that targeted interventions before RTS may have been warranted to improve maximum strength, power, and plyometric ability^18,23,51^ to enhance the modulation of the Stretch Shortening Cycle^11,25^ and to increase general strength as well as knee flexion force generation capacity.

Similar power (double-leg CMJ: relative peak power = 43.1 W/kg, jump height = 29.1 cm) and reactive strength (double-leg CMJ: RSImod = 0.33) characteristics were displayed by player 20 (25th percentile; TSA score = −0.68; 21 years old; 174 cm; 80 kg; bone–patellar tendon–bone graft; 8.5 months after surgery) (Figures 1 and 3). Low bilateral relative peak knee extension strength values (involved limb = 2.15 N·m/kg, uninvolved limb = 2.84 N·m/kg) were also shown compared with the rest of the cohort. At approximately 3 months after RTS, he reported a bucket-handle medial meniscal tear in the ACL-reconstructed knee. This player could have benefited from targeted strength and power training, with a particular focus on restoring knee extension strength until at least normative values were reached (ie, 3.0 N·m/kg).^51^

Player 73’s TSA score was in the first tertile (69th percentile; TSA score = 0.49; 22 years old; 180 cm; 73 kg; bone–patellar tendon–bone graft; 10.2 months after surgery) (Figures 1 and 4), but he sustained a grade 1 hamstring strain injury in the uninvolved limb at around 4 weeks after RTS. He showed above average strength (relative peak knee extension strength = 3.49 N·m/kg, relative peak knee flexion strength = 2.03 N·m/kg), power (single-leg CMJ: relative peak power = 33.3 W/kg, jump height = 17.9 cm), and reactive strength (single-leg CMJ: RSImod = 0.27) qualities in the uninvolved limb, but these were not matched by the ACL-reconstructed limb (with the exception of relative peak knee extension strength = 3.38 N·m/kg). In the absence of details to examine his soccer training program, match schedule, and training volume, it may be speculated that reduced physical qualities in his ACL-reconstructed limb (single-leg CMJ: relative peak power = 30.1 W/kg, jump height = 14.9 cm, RSImod = 0.19; relative peak knee flexion strength = 1.78 N·m/kg) could have resulted in abnormal sagittal mechanics of the ACL-reconstructed limb at the stance phase of running commonly found at RTS,^33^ requiring compensatory strategies and creating higher stress on the hamstring muscles in the contralateral limb. Therefore, it may have been prudent to develop single-leg posterior chain strength, and plyometric and power training can be accompanied by running drills to facilitate the integration of the newly acquired qualities into the cyclical motion of running and sprinting.^46^

### Limitations

The tests included in this study were limited to those routinely used to assess an athlete’s current level of physical capacity related to ACL research.^24^ However, the TSA can and should encompass a broader range of aspects (hip and ankle strength, aerobic capacity, psychological readiness, agility, etc). While the TSA score provides an overall indication of general sport readiness, it is also prudent to examine movement strategies that may be associated with the reinjury risk.^16,17^ Therefore, analysis of the athlete’s kinetics and kinematics during the execution of tasks is also advised. Similarly, we only extracted peak torque values from our isokinetic strength assessment, and further angle-specific analysis to more accurately identify residual deficits could be recommended.^13,41^ Clinicians should also consider psychological readiness^27^ and ensure that the requisite training volume representative of a player’s sports demands has been met in a progressive manner throughout his or her RTS journey.^42^ Although it may be assumed that training and game exposure among our players were similar, detailed access to exposure data was not available and should be considered in further studies.

Our data were also limited to adult male soccer players. However, TSA results are related to the cohort, sport, and tests assessed and thus could be generalized to pediatric, adolescent, and female athletes. Finally, although only commonly available spreadsheet software (eg, Excel) is needed for the integration of the TSA in clinical practice, contextualization of players’ TSA scores at the time of RTS after ACL reconstruction with those of matched controls requires a sufficient number of healthy players’ test scores be readily available. Therefore, it is recommended to routinely administer RTS tests encompassing strength, power, and reactive strength qualities each season across the largest possible number of players (ideally teammates). This allows benchmark data to be stored (including preinjury values) and used to generate the TSA score. Owing to seasonal variations in strength and power characteristics, periodic assessments at later time points (>4 months after RTS) are advised to further explore implications of the TSA with the long-term risk of subsequent injuries.^1^ Because of the low number of players sustaining a subsequent injury, it was only possible to observe trends in frequency distributions and use visual inspection. Future research may examine if lower TSA scores are associated with an increased injury risk in larger athletic cohorts.

## Conclusion

The findings of the current study indicate that a composite score (TSA) including strength, power, and reactive strength characteristics differed between elite soccer players at the time of RTS after ACL reconstruction and healthy matched controls. The TSA can be used to determine physical readiness and discriminate players’ status and be readily utilized by health care and sports professionals to identify the achievable targets needed during rehabilitation for the restoration of physical performance relative to peers competing at the same level. Preliminary data indicate that doing so has positive implications for lowering the risk of subsequent injuries, but further research is required to more clearly elucidate these findings in larger cohorts using statistical modeling.
